# Clusterin modulates transdifferentiation of non-small-cell lung cancer

**DOI:** 10.1186/s12885-017-3649-y

**Published:** 2017-09-27

**Authors:** Runsen Jin, Xingshi Chen, Dingpei Han, Xiaoying Luo, Hecheng Li

**Affiliations:** 1 Department of Thoracic Surgery, Ruijin Hospital, Shanghai Jiao Tong University School of Medicine, 197 Ruijin 2nd Road, Shanghai, 200025 People’s Republic of China; 2 State Key Laboratory of Oncogenes & Related Genes, Shanghai Cancer Institute, Renji Hospital, Shanghai Jiao Tong University School of Medicine, No.25/Ln2200, XieTu Road, Shanghai, 200032 People’s Republic of China

**Keywords:** Clusterin, Transdifferentiation, Lung cancer, Adenocarcinoma, Squamous cell carcinoma

## Abstract

**Background:**

Secreted clusterin (sCLU), a 75–80 kDa disulfide-linked heterodimeric protein, plays crucial roles in various pathophysiological processes, including lipid transport, tissue remodeling, cell apoptosis and reproduction. Our previous studies demonstrated that sCLU could influence cell apoptosis, proliferation, and invasion of non-small cell lung cancer (NSCLC) cells.

**Methods:**

In this study, clusterin’s function in regulating transdifferentiation of NSCLC cells was investigated. In addition, we examined the correlation between clusterin and clinicopathological features of lung cancer.

**Results:**

We found that clusterin was increased in lung adenocarcinoma tissues and decreased in lung squamous cell carcinoma tissues through immunohistochemical technique. In cultured lung adenocarcinoma cell lines, clusterin addition could increase SP-C protein expression in 2.75-fold, and decrease p63 protein expression in 0.65-fold (1.54 to 1). And also clusterin addition could increase SP-C mRNA expression in 4.05-fold, decreased p63 mRNA expression in 0.51-fold.

**Conclusions:**

Our study demonstrated that clusterin could promote EMT and influence transdifferentiation from lung squamous cell carcinoma to lung adenocarcinoma. However, we found that clusterin expression have no correlation with malignance associate clinicopathological data. Our study may help to further elucidate the development and progression of NSCLC, also it may contribute to the research of therapies targeting sCLU.

## Background

Lung cancer is the leading cause of cancer-related deaths and remains a formidable health burden throughout the world [1]. Generally, it can be divided into two types: non-small-cell lung cancer (NSCLC) and small-cell lung cancer (SCLC). As the major type, non-small cell lung cancer (NSCLC) is further classified by pathological characteristics into adenocarcinoma (ADC, 48%), squamous cell carcinoma (SCC, 28%) and large cell carcinoma (24%) [2]. ADC and SCC, which represent for more than 70% of lung cancer, have many differences in origins, treatments and prognosis. Interestingly, a special type of lung cancer was identified with the characters of ADC and SCC, called adeno-squamous cell carcinoma (AD-SCC). In addition, current studies have indicated that lung cancer cells can trans-differentiate between ADC and SCC [3, 4]. Coincidently, another kind of transdifferentiation, epithelial-mesenchymal transition (EMT), has been detected in NSCLC for several years [5], which is supposed to be related with cancer invasion, metastasis and drug resistance [6, 7]. Various proteins and signalling pathways have been demonstrated to closely correlate with EMT in NSCLC [8], including some molecular chaperones.

Clusterin(CLU), also known as apolipoprotein J, is a 75–80 kDa disulfide-linked heterodimeric protein, overexpressing in many cancers such as prostate cancer, lung cancer, breast cancer, etc. [9]. In human, there are two isoforms of CLU: secretory CLU protein (sCLU) (75–80 kDa) and nuclear CLU protein (nCLU) (55 kDa), they play different roles in process of cell growth apoptosis. Overexpression of sCLU protects the cell from apoptosis induced by cellular stress, such as chemotherapy or radiotherapy [10, 11]. It is reported that inhibition of CLU can increase the sensitivity of prostate cancer chemotherapy [12]. Another study also demonstrated that anti-sCLU antibody can inhibits TGF- induced EMT of liver cancer [13]. However, the relationship between CLU and NSCLC, especially the transdifferentiation of lung cancer cells was unclear and require elucidation.

In the current investigation, we examined whether sCLU could influence transdifferentiation of NSCLC cells. Also, we examined the correlation between sCLU and clinicopathological features of lung cancer. Our study demonstrated that sCLU could promote EMT and transdifferentiation lung squamous cell carcinoma to lung adenocarcinoma, which may reveal some underlying molecular mechanisms of NSCLC tumourigenesis and progression.

## Methods

### Human lung cancer specimen collection

All the specimens of human lung ADC and SCC were collected in Ruijin Hospital Shanghai Jiaotong University School of Medicine from 2011 to 2014, with patient written consents and the approval from Ruijin Hospital Ethics Committee. All tumor specimens were harvested at the time of surgical resection. Clinical features, including age at diagnosis, smoking history, gender, were collected. Seventy-five lung ADC samples and SCC were used for immunohistochemistry analysis.

### Immunohistochemical staining and scoring

Immunohistochemical staining was performed flowing standard protocols [14]. Briefly, the paraffin-embedded slides were deparaffinised in xylene and rehydrated using alcohol washes of increasing concentrations, then washed with PBS three times for 5 mininuts each time. For antigen retrieval, paraffin-embedded sections were microwave-treated in a moist chamber containing Tris-EDTA solution at room temperature, washed with PBS, and then immersed in 3% H_2_O_2_ solution at room temperature to abrogate endogenous peroxidase activity. The slides were incubated in 5% BSA to block non-specific binding of antibody for 20 min, then incubated with 1:500 diluted primary antibody overnight at 4 °C. After PBS washes, the slides were incubated with1:1000 diluted secondary antibody for 45–60 min at room temperature, followed by PBS washes again. The slides were visualized by employing EliVision TM plus two-step system with diaminobenzidine (DAB), stained with hematoxylin staining solution, dehydrated with graded alcohol series, covered-slipped with neutral balsam. The stained slides were observed and scored under a light microscope by pathologists, according to percentage of the cells of interest staining positive (0%: 0;1 ~ 29%: 1; 30 ~ 69%: 2; and ≥70%: 3). We define IHC intensity as the following formula:

$$ IHC\  intensity=\frac{Score of each patient}{Average score} $$

### Cell culture

NSCLC cell lines A549, provided by Shanghai Cancer Institute, were grown in the medium of DMEM containing 10% fetal bovine serum (FBS). Clusterin fragment was amplified from human lung cancer cDNA, purifying sCLU proteins from a eukaryotic expression system. Constructed eukaryotic expression vectors of pRAG5-flag-sCLU were transfected into HEK-293F cells. The sCLU proteins were purified by affinity chromatography, then detected using Flag and Clusterin antibody. We add sCLU and BSA into culture medium, acquiring a concentration gradient of sCLU. All experiments were performed in triplicates.

### Real-time RT-PCR (qRT-PCR)

Quantitative real-time PCR was performed as described previously [15]. We use TRIzol reagent (Invitrogen, Carlsbad, CA) to extract total RNA from cells and tissues, according to the manufacturer’s protocol. cDNA was reverse-transcribed from 1 μg of RNA using the SYBR®Prime ScriptTM RT-PCR kit (Takara Biochemicals, Tokyo, Japan), and the reactions were performed on an ABI PRISM®7900HT Real-Time PCR System. The thermal cycling conditions were as follows: an initial step at 95 °C for 15 s followed by 40 cycles of 95 °C for 5 s and 60 °C for 30 s. Each experiment was performed in a 20-μl reaction volume containing 10 μl of SYBR® Prime Ex TaqTM II (2×), 0.8 μl of forward primer and reverse primer (10 μM each), 0.4 μl of ROX Reference Dye or Dye II (50×), 2 μl of cDNA, and 6 μl of H_2_O. Primers used for qRT-PCR analysis were as follows: SP-C, 5′- cctgagtgagcacctggtta-3′ (forward) and 5′- tcaagactggggatgctctc-3′ (reverse); p63, 5′- gcagttgtgttggagggatg-3′ (forward) and 5′-gcttcgtaccatcaccgttc-3′ (reverse); β-actin, 5′-cccgccgccagctcaccatgg-3′ (forward) and 5′-aaggtctcaaacatgatctgggtc-3′ (reverse). β-actin was used as an internal control. The quantification of the mRNA was calculated using the comparative Ct (the threshold cycle) method according to the following formula: Ratio = 2-∆∆ct = 2-[∆Ct(sample)-∆Ct(calibrator)], where ∆Ct is equal to the Ct of the target gene minus the Ct of the endogenous control gene (β-actin).

### Western blot analysis

Western blot analysis was performed by established protocols [15]. Proteins were separated by SDS-polyacrylamide gel electrophoresis. Following electrophoretic separation, the proteins were transferred to a polyvinylidene fluoride membrane (Bio-Rad, Hercules, CA), where they were blocked with 5% non-fat milk and then stained with the following antibodies: the epithelial cell marker ZO-1 (1:200) and E-cadherin (1:1000), the mesenchymal cell marker Vimentin (1:2000), β-actin (1:2000, Santa Cruz Biotechnology, Santa Cruz, CA), and GAPDH (1:10,000; Kang-Chen Bio-tech Shanghai, China), the ADC marker SP-C (1:2000), the SCC marker p63 (1:2000). The quantification of Western Blot was exerted by Imagine J software (NIH, USA).

### Statistical analysis

Statistical analyses were performed with an SPSS software program (Version 22.0; SPSS Inc., Chicago, IL, USA). The results are presented as the mean ± S.D. Student’s t-test or one-way analysis of variance was used for comparing differences between two groups. The significance of proteins expression at different stages of lung cancer was identified using Mann–Whitney U-test. A two-tailed χ^2^ test was used to determine the association between protein expression and clinicopathological characteristics.

## Results

### Clusterin IHC intensity is up-regulated in lung adenocarcinoma and down-regulated in lung Squamous Cell Carcinoma

**Fig. 1:**
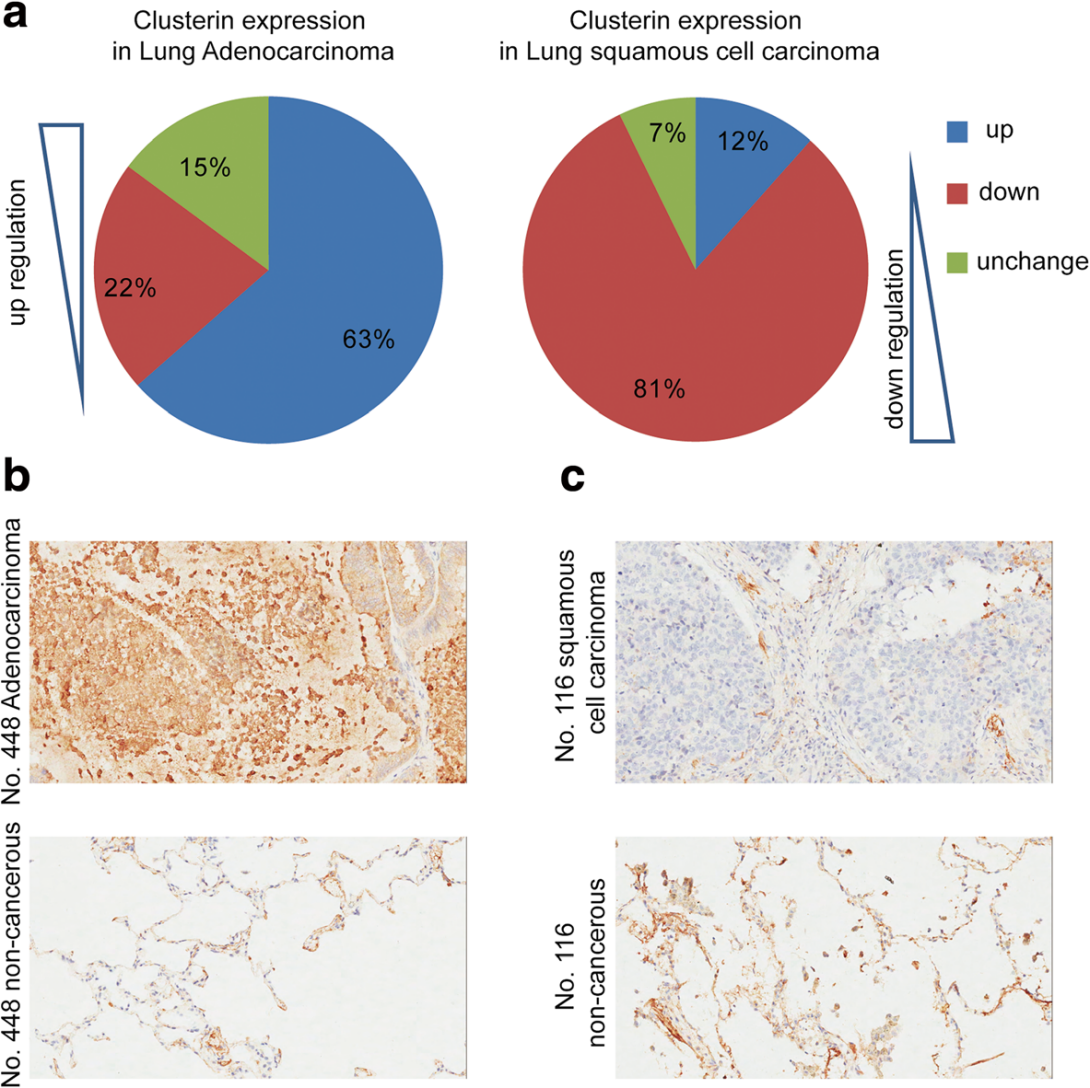
IHC assay demonstrates that clusterin IHC intensity are up-regulation in lung adenocarcinoma and down-regulation in lung Squamous Cell Carcinoma. Human lung cancer specimens were prepared as described in the Methods and then IHC was performed. The IHC intensity was confirmed by pathologist, and the IHC intensity were compared in lung cancer and its adjacent non-cancerous tissues, and combined the positive rate of IHC in cells (**a** & **b** & **c**)

To confirm the Clusterin expression in lung cancer, we detect clusterin expression in one lung adenocarcinoma tissue array slide and one lung squamous cell carcinoma tissue array slide (75 pairs each) by IHC assay. The clusterin intensity were increased in 63% of the samples (47/74, Fig. 1a, one piece of non-cancerous tissue lose), decreased in 22% of the samples (16/74, Fig. 1a), and unchanged in 15% of the samples (11/74, Fig. 1a). On the other hand, the clusterin intensity were increased in 12% of the samples (8/69, Fig. 1a, six pieces of non-cancerous tissue lose), decreased in 81% of the samples (56/69, Fig. 1a), and unchanged in 7% of the samples (5/69, Fig. 1a). The IHC intensity was confirmed by pathologists, who didn’t know which group the samples belonged to in advance, and the IHC intensity were compared in lung cancer and its adjacent non-cancerous tissues, and combined the positive rate of IHC in cells.

The data show clusterin were high-expression in lung adenocarcinoma (compare to its adjacent non-cancerous tissues), low-expression in lung squamous cell carcinoma (compare to its adjacent non-cancerous tissues). So clusterin show contrary expression profile in two categories of lung cancer.

### Correlation between clusterin IHC intensity and clinicopathological data in lung cancer

We also analysed the correlations between clusterin IHC intensity and multiple clinicopathological parameters.

In the cohort of lung adenocarcinoma, we found that clusterin IHC intensity do not have a significance difference between male and female group (*p* = 0.8382), the age above 60 and below 60 group (*p* = 0.6601), tumor size above 3 cm and below 3 cm group (*p* = 0.7610), furthermore, clusterin IHC intensity also do not have a significant difference in malignance associate clinicopathological data in lung adenocarcinoma (for example, clusterin IHC intensity have no significance difference in having positive lymph node and negative lymph node group (*p* = 0.1553), in different TNM stages (*p* = 0.5883), and in survival time (*p* = 0.6917)) (Table 1).

**Table 1:** Clinical information of 75 lung adenocarcinoma and clusterin IHC intensity

Parameters	Number of cases	Clusterin IHC intensity	*P*-value
Total case number	75	1.0000 ± 0.0623	
Gender			
Male	40	0.9880 ± 0.0888	0.8382
Female	35	1.0140 ± 0.0881	
Age (yr)			
< =60	39	0.9640 ± 0.1013	0.6601
> 60	36	1.0210 ± 0.0779	
Tumor size (cm)			
< =3	37	1.0190 ± 0.0760	0.7610
> 3	38	0.9811 ± 0.0992	
Lymphnode status(*n* = 71)			
Positive	36	0.8858 ± 0.0773	0.1553
Negative	35	1.0700 ± 0.1016	
TNM stage (*n* = 75)			
I	21	1.0380 ± 0.1056	0.5883
II	39	0.9410 ± 0.0761	
III, IV	15	1.0990 ± 0.1949	
Survival time (mon)			
< =40	40	1.0230 ± 0.0992	0.6917
> 40	35	0.9732 ± 0.0719	

In the cohort of lung squamous cell carcinoma, we also do not find that the clusterin IHC intensity have significance difference malignance associate clinicopathological data (Table 2).

**Table 2:** Clinical information of 71 lung squamous cell carcinoma and clusterin IHC intensity

Parameters	Number of cases	Clusterin IHC intensity	*P*-value
Total case number	71	1.0000 ± 0.0788	
Gender			
Male	67	1.0150 ± 0.0829	0.4384
Female	4	0.7481 ± 0.1090	
Age (yr)			
< =64	36	1.1520 ± 0.1308	0.0429*
> 64	35	0.8303 ± 0.0810	
Tumor size (cm)			
< =3.5	37	0.9262 ± 0.1120	0.3821
> 3.5	34	1.0690 ± 0.1142	
Lymphnode status			
Positive	36	1.0780 ± 0.1380	0.5850
Negative	35	0.9860 ± 0.1028	
TNM stage			
I	14	1.0420 ± 0.2009	0.8503
II	39	1.0160 ± 0.1016	
III, IV	18	0.9194 ± 0.1716	
Survival time (mon)			
< =60	36	1.0200 ± 0.1165	0.7968
> 60	35	0.9792 ± 0.1073	

* statistical difference

These data show that clusterin IHC intensity have no correlation with malignance associate clinicopathological data, and do not play critical role in the malignance of lung cancer in our cohorts.

### The level of clusterin in serum is a potential biomarker in lung cancer

**Fig. 2:**
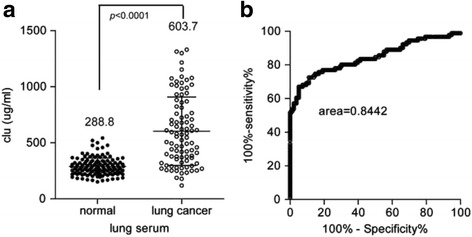
Scatter diagram shows that the level of clusterin in serum is a potential biomarker in lung cancer. The level of clusterin in the serum was detected in lung cancer patients and normal control. The results show that the level of clusterin is higher in lung cancer patient (**a**) and it could be a potential biomarker (**b**)

To further elucidate the significance of clusterin, we detect the level of clusterin in the serum of lung cancer patients. The data show that the level of clusterin in lung cancer patient is higher than the level of clusterin in normal control (Fig. 2a, 603.7 / 288.8, *p* < 0.0001). Furthermore, the level of clusterin in serum could be a potential biomarker of lung cancer (Fig. 2b, its ROC area = 0.8442).

We also analyzed the correlation between the level of clusterin in serum and the clinicopathological data. The data also show that the level of serum clusterin have no significance difference in different lymph node status patients (Table 3, *p* = 0.8142), as well as tumor size (Table 3, *p* = 0.4066). As a result, we conclude that the level of serum clusterin in lung cancer have no significance correlation with malignance associate clinicopathological data (although the level of serum clusterin have significance difference between TNM stages, *p* = 0.0013, Table 3).

**Table 3:** Clinical information of 91 lung cancer and level of clusterin

Parameters	Number of cases	Clusterin level (μg/mL)	*P*-value
Total case number	91	603.7 ± 31.97	
Gender			
Male	54	624.1 ± 44.98	0.4429
Female	37	573.8 ± 43.54	
Age (yr)			
< =60	50	584.0 ± 43.01	0.4990
> 60	41	627.7 ± 48.11	
Tumor size (cm)			
< =3.0	51	627.3 ± 40.93	0.4066
> 3.0	40	573.5 ± 50.86	
Lymphnode status(*n* = 88)			
Positive	40	591.2 ± 49.12	0.8142
Negative	48	606.3 ± 49.29	
TNM stage			
I	28	709.2 ± 61.32	0.0013*
II	53	516.1 ± 39.11	
III, IV	10	835.2 ± 91.84	

* statistical differencee

These data suggest that the level of serum clusterin could be a potential biomarker in lung cancer, but do not correlate with the malignance of lung cancer.

### Clusterin exert its influence in transdifferentiation between lung adenocarcinoma and lung squamous cell carcinoma

**Fig. 3:**
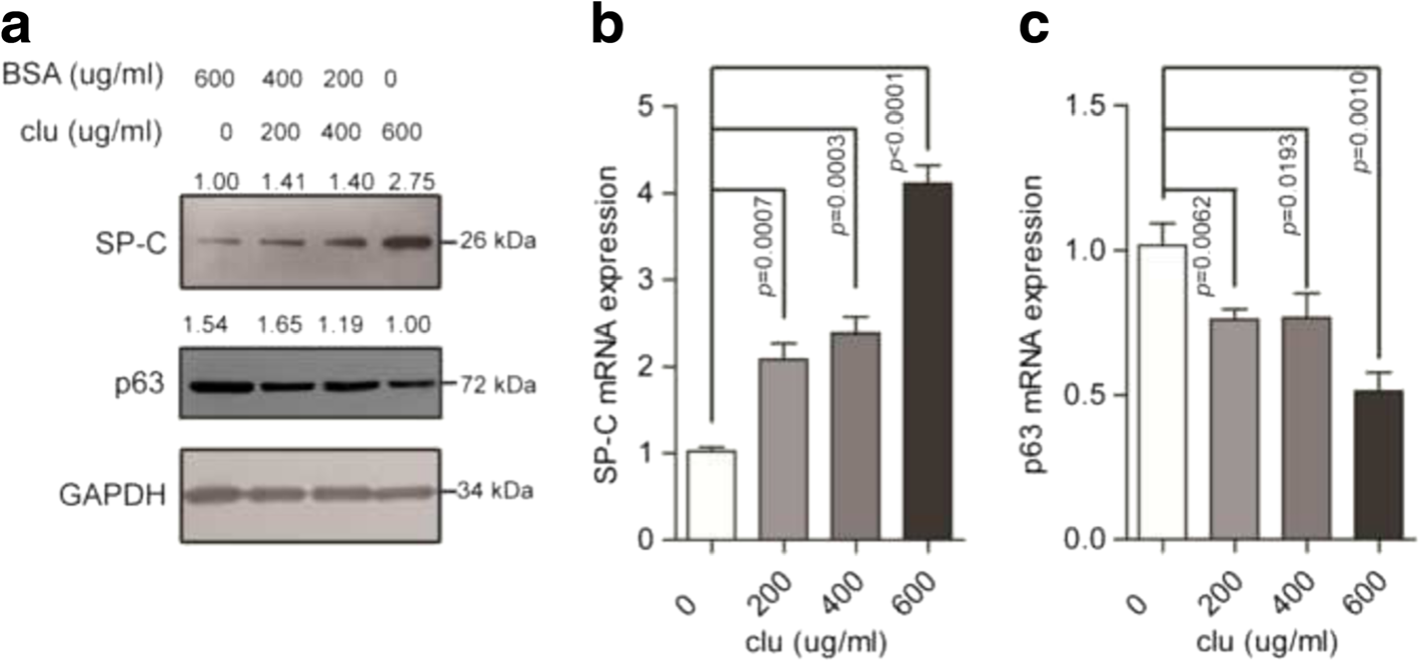
Western blot and qRT-PCR demonstrate that clusterin exert its influence in transdifferentiation between lung adenocarcinoma and lung squamous cell carcinoma. sCLU was added in concentration gradient, then, Western blot (**a**) and qRT-PCR (**b** & **c**) was performed as described in the Methods and the clusterin addition could increase SP-C protein expression in 2.75-fold, and decrease p63 protein expression in 0.65-fold (1.54 to 1). And also could increase SP-C mRNA expression in 4.05-fold, decreased p63 mRNA expression in 0.51-fold

In order to detect clusterin’s function in lung cancer differentiation, we confirmed the SP-C and p63 protein and mRNA levels in A549 cell lines. ADC mainly expresses type II pneumocyte marker pro-surfactant protein C (SP-C), and SCC expresses basal cell marker Trp63(p63). The clusterin addition could increase SP-C protein expression in 2.75-fold, and decrease p63 protein expression in 0.65-fold (1.54 to 1) (Fig. 3a). And it could also increase SP-C mRNA expression in 4.05-fold (Fig. 3b), decreased p63 mRNA expression in 0.51-fold (Fig. 3c).

These data indicated clusterin addition could up-regulate SP-C expression / down-regulate p63 expression, which suggest that it may influence transdifferentiation between lung adenocarcinoma and lung squamous cell carcinoma.

### Clusterin exert its influence in lung cancer epithelial-mesenchymal-like transition

**Fig. 4:**
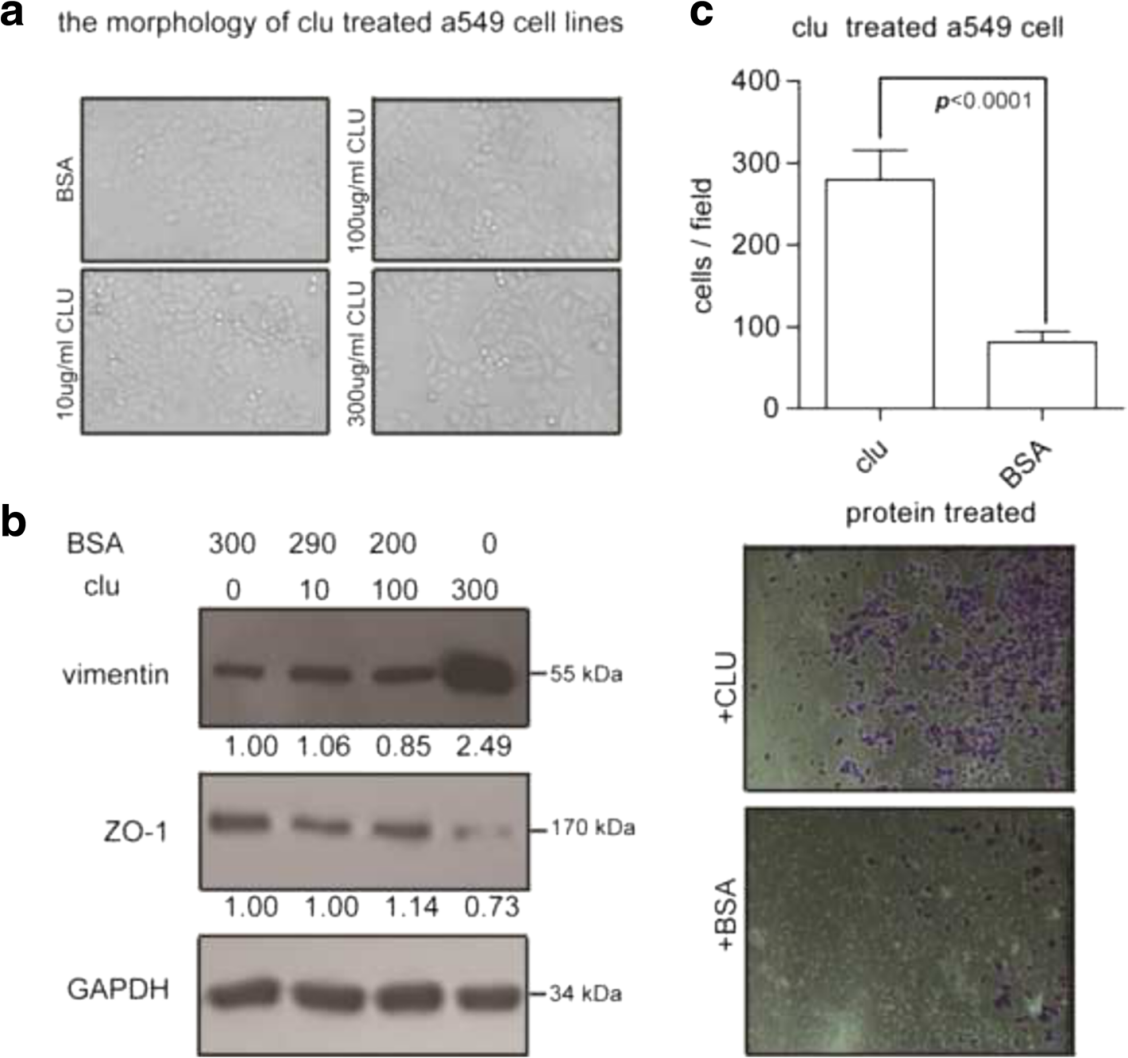
Cell morphology followed by western blot and transwell invasion assays demonstrate that clusterin exert its influence in lung cancer epithelial-mesenchymal transition. A549 cells were treated with sCLU in concentration gradient. Cell morphology (**a**) and density (**b**) were significantly altered. Transwell invasion assays (Corning, USA) were applied to A549 cells treated with sCLU or BSA according to manufacturer’s instruction (**c**)

Clusterin also exert another transdifferentiation function, epithelial-mesenchymal transition in other researches. The mesenchymal-like transition includes transition of cell morphology, surface markers, and motility. Firstly, we examined the morphology of A549 cells with altered clusterin addition concentration in medium. The experiment showed that the cell morphology was significantly altered with the increase of concentration (Fig. 4a). When the clusterin concentration increased to 300 μg/mL, the spindle-like cell morphology appears. And then we examined epithelial cell marker zonula occludens-1 (ZO-1) and mesenchymal cell marker vimentin expression levels through immunoblots. The data showed that altered clusterin concentration could change these markers’ expression. When the concentration of clusterin in medium increased to 300 μg/mL, the expression of epithelial cell marker ZO-1 increased, and the expression of vimentin, a mesenchymal cell marker, was decreased (Fig. 4b). Furthermore, we checked the cell motility with clusterin addition in medium. When 300 μg/mL clusterin add in the down space of chamber, the cell invasion is increased to 3.56-fold (Fig. 4c).

These data showed that increased clusterin concentration in medium could promote A549 cell line transition to mesenchymal-like cell.

## Discussion

A variety of studies have shown the important role of clusterin in regulating cancer cell apoptosis, tumorigenesis, and tumor progression [16–19]. However, complete understanding of its function and mechanism of action remains an important research goal. This investigation demonstrates a unique role for clusterin in influencing NSCLC cells transdifferentiation. To be more specific, clusterin could promote transdifferentiation from lung squamous cell carcinoma to lung adenocarcinoma and EMT of lung cancer cell. Furthermore, we demonstrated that the level of clusterin in serum could be a potential biomarker in lung cancer. This is the first time that clusterin has been shown to modulate lung cancer cell transdifferentiation.

Transdifferentiation, also called metaplasia, means conversion of one differentiated cell type into another. It is a complex process that one mature somatic cell transforms into another mature somatic cell without undergoing an intermediate pluripotent state or progenitor cell type [20]. Tumors in various organs, including lung, have shown their phenotypic plasticity [21] and pathological heterogeneity. Obviously, researches on these properties of tumor offers a potential explanation of tumourigenesis, proliferation, metastasis and drug resistance, which will contribute to the development of novel cancer therapeutic strategies. There have been researches which showed the capacity of glioblastoma stem cells transdifferentiating into endothelial cells [22, 23]. The histopathological types of lung cancer has been established, however, the stability of phenotype in each kind of lung cancer and the convertibility between the tumour types remain unclear. Indeed, several researches have focused on this. Previous studies have demonstrated that lung cancer cells can trans-differentiate between ADC and SCC [3, 4]. One recent study has revealed the genotypic and histological transition of EGFR-mutant NSCLC into SCLC after molecular targeted therapy [24]. In the current study, we demonstrated that clusterin are up-regulation in lung adenocarcinoma and down-regulation in lung squamous cell carcinoma (Fig. 1). Most importantly, we show that clusterin could up-regulate SP-C expression / down-regulate p63 expression (Fig. 3). Collectively, our study indicated clusterin could influence transdifferentiation from lung squamous cell carcinoma to lung adenocarcinoma, this potentially associated with lung cancer origination and progression.

Epithelial-mesenchymal transition(EMT), another kind of transdifferentiation, has been found to be critical in tumor local invasion and distant metastasis [25, 26], endowing cells with some properties of cancer stem cell [27]. Recent study also revealed that EMT is associated with lung cancer chemoresistance [28]. In the process of EMT, the most notable characteristic is down-regulation of epithelial markers’ expression like ZO-1 and up-regulation of mesenchymal markers’ expression like vimentin, which leads to numerous phenotypic changes such as the loss of cellular adhesion and polarity and the acquisition of migratory and invasive properties [29]. A previous study reported that clusterin silencing in human lung adenocarcinoma cells induces a mesenchymal-to-epithelial transition [30]. We found that increasing concentration of clusterin in medium can result in a spindle-like cell morphology of A549 cells (Fig. 4a). In addition, we also demonstrated the down-regulation of ZO-1 expression and up-regulation of vimentin expression in clusterin incubated A549 cells. This indicates that clusterin induce NSCLC cell EMT, which probably has a close relation with tumor metastasis and drug resistant.

In the current study, we also analyzed clusterin expression and clinicopathological data in lung cancer. Interestingly, we found that clusterin IHC intensity have no correlation with malignance associate clinicopathological data. This result is kind of contradiction to our initial hypothesis. For the reason, we suppose that clusterin could also protect normal cells from senescence [31], which is beneficial for systemic situation. Also, according to Park et al.’s review, clusterin can promote survival due to its cardioprotective, antifibrosis, and antidiabetes function [32]. On the other hand, data show that the level of clusterin in lung cancer patient is significantly higher than normal control, with acceptable sensitivity and specificity, which indicate that clusterin is a potential new biomarker of NSCLC. Admittedly, more researches are needed to confirm the diagnostic and prognostic role of clusterin in NSCLC.

Of note, the underlying mechanisms of transdifferentiation is intricacy, which involves a lot of signalling pathways such as NFκB, wnt/β-catenin, ERK, etc. [8]*.* As for the transdifferentiation between ADC and SCC, a recent study has demonstrated that YAP can inhibit squamous transdifferentiation of Lkb1-deficient lung adenocarcinoma [3, 4]. Importantly, how clusterin influence the transdifferentiation between ADC and SCC remains a mystery. Thus, more efforts are required to uncover the specific process of clusterin induced transdifferentiation.

## Conclusion

In conclusion, we demonstrate that clusterin can influence transdifferentiation from lung squamous cell carcinoma to lung adenocarcinoma and promote EMT in NSCLC cells. Moreover, clusterin is an independent diagnostic biomarker of NSCLC.

## Abbreviations

ADC: Adenocarcinoma
AD-SCC: Adeno-squamous cell carcinoma
EMT: Epithelial-mesenchymal transition
IHC: Immunohistochemistry
nCLU: Nuclear clusterin
NSCLC: Non-small cell lung cancer
SCC: Squamous cell carcinoma
SCLC: Small cell lung cancer
sCLU: Secreted clusterin
